# Application of a novel lytic *Jerseyvirus* phage LPSent1 for the biological control of the multidrug-resistant *Salmonella* Enteritidis in foods

**DOI:** 10.3389/fmicb.2023.1135806

**Published:** 2023-04-05

**Authors:** Rashad R. Al-Hindi, Mona G. Alharbi, Ibrahim Alotibi, Sheren A. Azhari, Khloud M. Algothmi, Ahmed Esmael

**Affiliations:** ^1^Department of Biological Sciences, Faculty of Science, King Abdulaziz University, Jeddah, Saudi Arabia; ^2^Health Information Technology Department, Applied College, King Abdulaziz University, Jeddah, Saudi Arabia; ^3^Botany and Microbiology Department, Faculty of Science, Benha University, Banha, Egypt; ^4^Nebraska Center for Virology, University of Nebraska–Lincoln, Lincoln, NE, United States

**Keywords:** bacteriophages, *Salmonella* Enteritidis (*S*. Enteritidis), multi-drug resistant (MDR), biofilms, biocontrol

## Abstract

Non-typhoidal *Salmonella* is the tremendously predominant source of acquired foodborne infection in humans, causing salmonellosis which is a global threat to the healthcare system. This threat is even worse when it is combined with the incidence of multidrug-resistant *Salmonella* strains. Bacteriophage therapy has been proposed as a promising potential candidate to control a diversity of foodborne infective bacteria. The objective of this study designed to isolate and characterize lytic phages infecting zoonotic multi-drug resistant and strong biofilm producer *Salmonella enterica* serovar Enteritidis EG.SmE1 and then apply the isolated phage/s as a biocontrol agent against infections in ready-to-eat food articles including milk, water, apple juice, and chicken breasts. One lytic phage (LPSent1) was selected based on its robust and stable lytic activity. Phage LPSent1 belonged to the genus *Jerseyvirus* within the *Jerseyvirinae* subfamily. The lysis time of phage LPSent1 was 60 min with a latent period of 30 min and each infected cell burst about 112 plaque-forming units. Phage LPSent1 showed a narrow host range. Furthermore, the LPSent1 genome did not encode any virulence or lysogenic genes. In addition, phage LPSent1 had wide pH tolerance, prolonged thermal stability, and was stable in food articles lacking its susceptible host for 48 h. *In vitro* applications of phage LPSent1 inhibited free planktonic cells and biofilms of *Salmonella* Enteritidis EG.SmE1 with a lower occurrence to form phage-resistant bacterial mutants which suggests promising applications on food articles. Application of phage LPSent1 at multiplicities of infections of 100 or 1000 showed significant inhibition in the bacterial count of *Salmonella* Enteritidis EG.SmE1 by 5 log_10_/sample in milk, water, apple juice, and chicken breasts at either 4°C or 25°C. Accordingly, taken together these findings establish phage LPSent1 as an effective, promising candidate for the biocontrol of MDR *Salmonella* Enteritidis in ready-to-eat food.

## Introduction

*Salmonella enterica*, a gram-negative facultative intracellular rod-shaped bacterium that is a member of the family *Enterobacteriaceae*, is one of the major widespread foodborne pathogens causing diseases and death worldwide (WHO, 2017). *Salmonella enterica* has approximately more than 2,600 serovars and roughly all are pathogenic (Porwollik et al., 2004; Gal-Mor, 2019) of which *S*. Typhi are host-specific, while other serovars like *S*. Typhimurium and *S*. Enteritidis are generalists. *S*. Typhi is associated with typhoid fever, Non-typhoidal *Salmonella* species (NTS), including *S*. Enteritidis and *S*. Typhimurium, mainly cause gastrointestinal infection which may lead to hospitalization and death (Musyoka et al., 2018).

Globally, NTS are the extremely prevalent cause of acquired foodborne infection in humans, causing salmonellosis (Esmael et al., 2021b). Salmonellosis is transmitted in humans through the oral-fecal route by the consumption of contaminated food either of animal origin (milk, meat, poultry, and eggs) or green vegetables contaminated by manure (Youssef et al., 2021). The infection symptoms include diarrhea, fever, vomiting, abdominal crumbs as well many other symptoms which arise one or two days after infection and can be persisted for a week (Wilson and Wilson, 2021). Although symptoms of salmonellosis are mild, and patients normally recover with no therapy, in some cases, it resulted in dehydration that may become severe and lead to hospitalization and death in children and immunocompromised patients (Musyoka et al., 2018). NTS have resulted in persistent infection in sub-Saharan Africa that showed bacteremia and septicemia with a mortality rate of ∼21% (Haselbeck et al., 2017). The World Health Organization (WHO) has declared that NTS results annually in about 94 million hospitalizations and 155,000 deaths worldwide (Majowicz et al., 2010). According to the Centers for Disease Control and Prevention (CDC), there are 23,000 illnesses and about 450 deaths caused by NTS species every year in the United States.

In poultry, *Salmonella* is identified to be present asymptomatically in the gastrointestinal tracts or generate enteric infection symptoms (Pandey et al., 2021). As a result, the disease remains undetected, and its appearance is related to the human consumption of contaminated food products (Foley et al., 2008). NTS-infected animals can spread the infection through contaminated feeds, the environment, or by direct contact with another infected animal (Atterbury et al., 2007). NTS infections are impacted by two factors: its broad host range and the presence of multi-drug resistance (MDR).

MDR is the antimicrobial resistance displayed by the bacteria to at least one agent in three or more antimicrobial categories. Some *Salmonella* strains encode several antimicrobial resistance genes that confer an MDR trait against more than one antibiotic. Lately, the occurrence of MDR *Salmonella* serovars has boosted the malfunction of antibiotic therapies (Medeiros et al., 2011; Agyare et al., 2019). Over several decades, the consumption of low doses of antibiotics in the poultry industry was a general practice (Moore and Evenson, 1946) not only for prophylactic or therapeutic concerns but, also, to promote growth (Waibel et al., 1954; Libby and Schaible, 1955), as and this improper use causes the emergence of MDR. The used antibiotics could not kill the whole gut bacteria, and certain resilient strains might survive and turn out to be resistant. Eventually, the resistant bacteria pass their antibiotic-resistant genes on to other susceptible bacteria.

Depending on the country’s economy, degree of development, livestock farming, and types of animals, antibiotics usage as a growth promoter varies in form and scope (Archawakulathep et al., 2014). The use of antibiotics to promote growth in poultry industry has been banned in the European Union (EU) since 2006, and in the US in 2017 (European Union, 2005; AccessScience Editors, 2017). On the 28^th^ of January 2022, the EU has taken the unprecedented step for sustainable antibiotic-free broilers production as the use of routinely fed a diet of antibiotics is now strictly banned and come into enforce. In the U.S. antibiotics. Although, poultry breeders in low- and middle-income countries, where antibiotic laws are not strictly enforced, still employ antibiotics intentionally to promote growth without any veterinary prescription (Maron et al., 2013). In Egypt, previous studies reported the incidence of MDR NTS contaminating different food articles (Diab et al., 2019; Youssef et al., 2021). Therefore, NTS human infections have become a major threat to healthcare systems around the world due to the annual increase in morbidity rates.

In food articles and industrial facilities, *Salmonella* frequently lives not only as free planktonic cells but also as sessile multicellular surface-associated forms known as biofilms. *Salmonella* virulence is attributed to the formation of biofilms, because bacteria in the complex biofilm communities are more resistant to antibiotics, resulting in a chronic *Salmonella* carries infection (Gonzalez-Escobedo et al., 2011; Gonzalez-Escobedo and Gunn, 2013; Zeineldin et al., 2023). Standard control procedures such as using special preservatives and heat treatment in liquid food are predominantly applied to control *Salmonella* load in food products and to reduce biofilms (Pérez-Díaz et al., 2008; Neetoo and Mahomoodally, 2014; Chylkova et al., 2017; Musyoka et al., 2018). Although these strategies are effective, the concern about the undesirable side effects given by the chemical stabilizers is discouraging (Pawlowska et al., 2012). In addition, heat treatment results in the degradation of important nutrients. Moreover, the application of antibiotics in food products is generally deterred because of non-specific antibacterial activity, long-term environmental stability, and the prevalence of MDR bacteria (Medeiros et al., 2011; Agyare et al., 2019).

On the contrary, due to bacteriophages’ nature of being obligate parasites, self-replicating ability, and host specificity, they are deemed as attractive antibacterial agents. Bacteriophages have received much more interest over antibiotics as a novel natural approach to control bacteria in food products and to ease their biofilms as well (Goodridge and Bisha, 2011; Matsuzaki et al., 2014; Lin et al., 2017; Esmael et al., 2021b). Moreover, phages do not cause hurt to eukaryotic cells (Kutter et al., 2010; McCallin et al., 2013) and no reports have described any bacteriophage infection in humans so far (Kutter et al., 2010; Keen et al., 2015). Therefore, phage treatment appears to be a good candidate as an antibacterial control in food. Different studies showed that phages were effective to control foodborne bacteria in different food materials (O’Flynn et al., 2006; Spricigo et al., 2013; Bao et al., 2015; Huang et al., 2018a; Esmael et al., 2021b; Alharbi et al., 2022).

Likewise, certain phages have been confirmed, by the FDA, as Generally Recognized as Safe (GRAS), being commercially available and used to combat *Salmonella* and their biofilms in food products, e.g., Armament, Salmonelex, SalmoFresh, and many others (Goodridge and Bisha, 2011; Sukumaran et al., 2015; Moye et al., 2018).

Earlier, five *Salmonella* enterica serovars were isolated from a poultry farm in Benha city, Qalubiya governorate, Egypt. Antibiotic susceptibility testing of these bacteria identified two MDR isolates (*S.* Enteriditis EG.SmE1 and *S*. Typhimurium EG.SmT3). In a previous study, we described the isolation, characterization, and application of three lytic *Salmonella* phages against *S*. Typhimurium EG.SmT3 to combat food-borne salmonellosis in various food articles. In this study, keeping in view the remarkable efficiency of lytic bacteriophages in limiting bacteria, we describe the efficacy of the newly isolated phage LPSent1 as a good candidate against *S.* Enteriditis EG.SmE1. Moreover, we investigate its efficacy to control zoonotic MDR *Salmonella* and their biofilms in ready-to-consume food articles including milk, water, Apple Juice, and chicken breasts.

## Materials and methods

### Bacterial origin and maintenance

Bacteria were preserved at −80°C in Brain-Heart-Infusion broth supplemented with 20% (v/v) glycerol. Fresh cultures were prepared prior to each experiment by inoculating a single colony into 5 mL tryptic soy broth (TSB, DifcoTM, USA) and incubating at 37°C for 16 h while shaking at 200 rpm.

### Antibiotic sensitivity test

The Kirby-Bauer disk diffusion method (Esmael et al., 2020, 2021a,2021b) was employed to check the antibiotic sensitivity pattern on Mueller-Hinton agar medium against a selection of thirteen antibiotics (Oxoid, Hampshire, UK): ampicillin (10 μg), amoxicillin (25 μg), ciprofloxacin (5 μg), amikacin (30 μg), gentamycin (10 μg), streptomycin (10 μg), tetracycline (30 μg), chloramphenicol (30 μg), aztreonam (30 μg), trimethoprim-sulfamethoxazole (25 μg), cephalexin (30 μg), cefoxitin (30 μg), and ceftriaxone (30 μg). Inhibition zones were measured and results were expounded according to the Clinical and Laboratory Standards Institute (CLSI) (Clinical and Laboratory Standards Institute, 2019). Tested bacteria was defined as MDR when acquired non-susceptibility to at least one agent in three or more antimicrobial categories.

### Quantitative evaluation of biofilm formation

The potential of biofilm production by the five *Salmonella* serovars in the current study was assessed as described previously (Stepanovic et al., 2007; Esmael et al., 2021a). A final concentration of 4 log_10_ CFU/mL of fresh *Salmonella* cultures was inoculated individually into Luria broth (LB) medium without NaCl in each well of a 96-well microtiter plate and incubated at 37°C for 24 h. Negative control wells containing *Salmonella*-free LB were involved. Consequently, the plates were emptied to remove the free planktonic cells, rinsed three times gently with phosphate-buffered saline (PBS), and air-dried. The residual attached bacteria were fixed with 98% methanol for 10 min., the methanol was decanted, and the plates were again allowed to air-dry. The fixed bacteria were then stained with 1% crystal violet for 45 min., rinsed three times gently with water to remove the excess crystal violet, then the stained cells were solubilized in 33% acetic acid.

A microplate reader (BMG LABTECH GmbH, Allmendgrun, Germany) was used to measure optical densities at 600 nm. To evaluate and categorize biofilm-generating serovars, an optical density cutoff (ODc) representing the background noise, or the threshold was used as described by Stepanovic et al. (2007), where ODc = average OD of negative control + (3 × SD of negative control). Bacteria were categorized into non-biofilm producers (OD ≤ ODc), weak biofilm producers (2 × ODc ≥ OD > ODc), moderate biofilm producers (4 × ODc ≥ OD > 2 × ODc), and strong biofilm producers (OD > 4 × ODc).

### Mitomycin-C induction to identify prophage-free *Salmonella*

The MDR and strong biofilm producer *Salmonella enterica* serovar Enteritidis EG.SmE1 was selected for bacteriophage isolation, purification, and propagation. Before environmental screening for lytic phages, *S*. Enteriditis EG.SmE1 was examined for lysogens (prophages) using chemical mitomycin C-mediated induction as described before (Klieve, 2005; Esmael et al., 2021a,b). Briefly, *S*. Enteriditis EG.SmE1 was grown in 5 mL of TSB medium until a mid-log phase (0.3 at 600 nm), then treated with mitomycin C (Sigma-Aldrich, St. Louis, MO, USA) at a final concentration of 0.2 μg/mL. The treated bacteria were then incubated at 37°C and bacterial growth was followed for 16 h by measuring the absorbance at OD_600nm_. At various time points (0.5 h, 1 h, 1.5 h, 2 h, 6 h, 10 h, 12 h, and 16 h), 500 μL aliquots of the treated bacteria were collected, cell debris was removed by centrifugation at 8,000 × *g* for 20 min, and then the lysates were passage through a 0.45 μm membrane filter. Collected lysates were then tested by spotting 10 μL from each lysate onto a lawn of *S*. Enteriditis EG.SmE1 and the plates were then incubated at 37°C for 24 h.

### Bacteriophage enrichment and isolation

Several environmental samples, including raw sewage water, agricultural farm ditches, and chicken feces, were collected and screened for bacteriophages against *S*. Enteriditis EG.SmE1 as illustrated before (Akhtar et al., 2014; Esmael et al., 2021b; Teklemariam et al., 2022). The collected water samples were processed to remove solids and cellular entities by centrifugation at 10,000 × *g* for 10 min and then were passage through 0.22 μm membrane filters (Mixed Cellulose Ester, MF-Millipore, Burlington, MA, USA). Chicken feces (10 g) were suspended in 50 mL TSB and were then administered in the same way as the water samples. Lytic phages were isolated from the processed samples via the enrichment method as described previously (Van Twest and Kropinski, 2009). In brief, 5 mL of sterile 2 × TSB medium pre-inoculated with 150 μL of fresh *S*. Enteriditis EG.SmE1 was mixed individually with 5 mL of the 0.22 μm-filtered samples, the tubes were then incubated at 37°C with continuous shaking at 200 rpm for 24 h. Enriched samples were later centrifuged at 10,000 × *g* for 10 min, the supernatants were collected and filtered using 0.22-μm membrane filters.

The presence of lytic phages was detected by spotting 10 μL of the enriched supernatants on lawns of *S*. Enteriditis EG.SmE1, then the plates were incubated at 37°C and were then observed for the formation of any lysis (Kropinski et al., 2009; Esmael et al., 2021b). To resuspend phages in the lysis zones, about 5 mL of filter-sterilized phage diluent (Salt-magnesium buffer) was added on top of the plate and gently shaken overnight at room temperature. Plate lysates were cleaned or purified through three rounds of single-plaque purification using the double-layer agar (DLA) method (Clokie and Kropinski, 2009). In brief, a single phage plaque was picked using sterile toothpicks, resuspended in 100 μL of SM buffer, and then kept overnight at room temperature. The process of isolating a single plaque and plating using the DLA method was repeated three times successively. The plate lysate was then collected, centrifuged at 4,000 × g for 5 min to remove any remaining debris, and stored at 4°C.

### Preparation and purification of a high−titer phage stock

The isolated phage was propagated and concentrated as mentioned earlier (Yamamoto et al., 1970; Esmael et al., 2021b). About 100 mL of a mid-exponential culture (0.3 at 600nm) of the indicator *S*. Enteriditis EG.SmE1 was inoculated with a purified phage suspension at a multiplicity of infection (MOI) of 1, then the mix was incubated for 24 h at 37°C while shaking at 200 rpm. Then the lysate was centrifuged at 4,000 × *g* for 5 min to remove the bacterial debris., free phages in the supernatant were then treated with 10% (*w/v*) Polyethylene glycol (PEG) 6,000 overnight at 4°C to permit phage precipitation on the PEG. Then the phage-PEG complex was pelleted by centrifugation at 10,000 × *g* for 30 min at 4°C, then the supernatant was gently poured off and the phage-PEG pellets were resuspended in SM buffer. A chloroform-based extraction method was deployed to release phages from the PEG particles as described previously (Yamamoto et al., 1970). Finally, the concentrated phages were filter-sterilized using 0.22-μm membrane filters, aliquoted into cryotubes, and stored at 4°C. The phage stock (PFU/mL) was titrated in triplicate using *S*. Enteriditis EG.SmE1 by the DLA method.

### Characterization of the isolated phage

#### Transmission electron microscopy

Morphological features of the isolated phage were determined by transmission electron microscope (A JEOL JEM-2100), located at the Electron Microscope Facility, Al-Mansoura University, Egypt. Ten microliters of a highly purified phage preparation (∼10^12^ PFU/mL) were transferred and fixed for 5 min. onto the surface of carbon-coated copper TEM film (Electron Microscopy Sciences) and were negatively stained with 2% (*w/v*) phosphotungstic acid, pH 7.2, for 1 min (Ackermann, 2012). After air-drying at room temperature for 1 h, the stained phage particles were observed under TEM.

#### Phage one-step growth kinetics

The growth curve of the isolated phage was evaluated as explained earlier (Esmael et al., 2021a,b). *Salmonella* Enteriditis EG.SmE1 (1 × 10^7^CFU/mL) was challenged (at 37°C while shaking at 200 rpm) with the isolated phage at an MOI of 1 for 5 min., then centrifuged at 5000 × *g* for 5 min to remove free unabsorbed phages in the supernatant. The pellet was then washed twice and then resuspended in 10 mL of sterile TSB, again reincubated at 37°C while shaking at 200 rpm. Aliquots of the resuspended bacteria were collected every 5 min for 1 hour post-infection (p.i.) and phage titers were counted using the double-layer agar (DLA) method.

#### Bacteriophage pH and temperature stability

The temperature stability of phage LPSent1 (10 log_10_ PFU/mL) was assessed at 30, 40, 50, 60, 70, and 80°C at pH 7.0 in an adjusted water bath incubator for either 30 min or 60 min as described before (Esmael et al., 2021a,b). For pH stability, phage LPSent1 (10 log_10_ PFU/mL) was diluted in SM buffer at different pH ranges (From 2-13) and incubated at 37°C for 24 h. Instantly after the thermal or pH treatment, serial dilutions of the treated phage were prepared, and the number of plaques was measured using the DLA assay. Phage residual percentage after each treatment was measured as follows:

Residual activity (%) = (phage count (PFU/mL) after treatment÷ Original phage count (PFU/mL)) × 100.

#### Phage DNA extraction, DNA sequencing, analysis, and assembly

LPSent1 genomic DNA was isolated using the direct plaque sequencing method as described before by Kot et al. (2014). The concentration of the isolated DNA was estimated using a NanoDrop ND-1000 UV-Vis spectrophotometer. Phage genomic DNA was stored at −20°C for further analysis. Phage DNA libraries were prepared using the Illumina DNA Prep kit and IDT 10 bp UDI indices, and sequenced on an Illumina NextSeq 2000, producing 2 × 151 bp reads. Demultiplexing, quality control, and adapter trimming were performed with bcl-convert (v3.9.3), and short reads were assembled with Unicycler (Wick et al., 2017). The assembled genome was annotated with prokka (v1.14.5) (Seemann, 2014) and the NCBI open-reading frame (ORF) finder search server. Putative functions of the identified coding sequences (CDSs) in each ORF were evaluated using the BLASTp search algorithm on the NCBI website. Detection of genes encoding tRNAs was evaluated using tRNAscan-SE v.1.3.1 (Lowe and Eddy, 1997). Assembled LPSent1 genomic DNA was searched for antimicrobial resistance genes using the ResFinder web service (Florensa et al., 2022). A genomic circular map of phage LPSent1 was built up and designed using CG view (Stothard and Wishart, 2005). The complete genome sequence of phage LPSent1 has been deposited in the GenBank database under the accession number OQ091358.

#### Construction of phylogenetic tree

MAFFT online server (Katoh et al., 2019) was used to align the nucleotide sequences of phage LPSent1 complete genome, and terminase large subunit gene against 13 phages in the *Jerseyvirus* genus, the formed alignment was then imported into the MEGA X program version 10.2.4 to construct the phylogenetic tree using the neighbor-joining method and 1000 bootstraps (Kumar et al., 2018).

#### Host range and efficiency of plating (EOP) analysis

The host range of the phage LPSent1 was revealed against a group of 18 *Salmonella* strains and 6 non-*Salmonella* strains (Supplementary Table 1) as described earlier (Clokie and Kropinski, 2009; De Melo et al., 2019) with some modifications. The first set of analyses was performed using undiluted phage preparation, aliquots of 10 μL of phage stock at a titer of 1 × 10^7^ PFU/mL were spotted, in triplicates onto the surface of the agar overlay of the tested bacteria. The plates were incubated for a period of up to 24 h at 37°C, then the plates were observed for the formation of inhibition lytic zones. The second set of analyses using bacterial isolates that showed clear or turbid zones was done using the same method described above, except using serially diluted (up to 10^7^ dilutions) phage preparations other than the undiluted stock. The plates were incubated as in the first set while this time, the plates were observed for the formation of typical plaques.

The efficiency of plating (EOP) of the isolated phage on the susceptible isolates was calculated as described before (Mirzaei and Nilsson, 2015; Huang et al., 2018a; Esmael et al., 2021b). A volume of 50 μL of the diluted phage stock (containing about 50 PFU) was mixed with 200 μL of the susceptible bacterial culture at the mid-exponential phase of growth, then the samples were assayed using the DLA method. EOP value was estimated, as follows:

EOP = number of PFUs on the tested bacteria/number of PFUs on *S.* Enteriditis EG.SmE1.

#### Determination of the frequency of bacteriophage insensitive mutants

The incidence of the development of bacteriophage-insensitive mutants (BIMs) was assessed as previously described (O’Flaherty et al., 2005). Phage LPSent1 was mixed with *S.* Enteriditis EG.SmE1 at an MOI of 10 and incubated for 20 min at 37°C, then, the suspension was diluted and platted using the DLA assay. Plates were incubated overnight at 37°C, any developed colonies confronted were counted and BIM frequency (Bacterial viable counts after phage infection divided by the original bacteria count) was calculated. Experiments were conducted in triplicate.

#### Efficacy of the isolated phage on planktonic cells

The bactericidal activity of phage LPSent1 against free cells of *S.* Enteriditis EG.SmE1 was determined at different MOIs as described before (Huang et al., 2018a; Esmael et al., 2021a,b). Briefly, 100 μL of *S.* Enteriditis EG.SmE1 culture (7 log_10_ CFU/mL) at the exponential phase was transferred into each well of a 96-well microtiter plate, then challenged with phage LPSent1 at an MOI of 0.1, 1, 5, or 10. Negative control wells contained phage-free *S.* Enteriditis EG.SmE1. Plates were incubated at 37°C in a static condition and bacterial growth was monitored for 6 h post-infection by measuring optical densities at 600 nm using a microplate reader (680 XR reader, Bio-Rad, Hercules, CA, USA).

#### Phage activity against established biofilm of *Salmonella* Enteriditis EG.SmE1

The efficacy of phage LPSent1 to inhibit biofilms of *S.* Enteriditis EG.SmE1 was measured by the quantitative colorimetric method as described before (Cerca et al., 2005) with some adaptations. To each well of a 96-well flat-bottomed microtiter plate, 100 μL of *S.* Enteriditis EG.SmE1 (final count of 6 log_10_ CFU/mL) was inoculated into sterile LB medium without NaCl for 24 h at 37°C, then the free unattached planktonic cells were gently aspirated. Then bacteria in the wells were challenged with phage LPSent1 at different MOIs (5, 10, 50). Phage LPSent1 was diluted in TSB and 100 μL aliquots of the diluted phage were added to each well at the corresponding MOI. Positive control wells received equivalent volumes of TSB. In a similar experiment, phage LPSent1 was added to wells every 6 h gently substituting the well-free materials (containing media, free phages, and planktonic *Salmonella* cells). The plates were further incubated under the same conditions, then the free materials of each plate were aspirated, and the biomass of the established biofilms was quantified. The plates were rinsed three times with phosphate buffer saline, then the plates were air-dried, fixed, stained with crystal violet, and analyzed as mentioned earlier. The absorbance (at 600 nm) was measured at 6, 12, 18, and 24 h using a microplate reader (BMG LABTECH GmbH, Allmendgrun, Germany).

#### Phage stability in foods

Stability of phage LPSent1 in milk, apple juice, water, and on chicken breast, experiments were conducted for two days as described previously (Huang et al., 2018b; Islam et al., 2019; Esmael et al., 2021b) at two different temperatures 4°C and 25°C representing food storage and food processing temperatures. Cow pasteurized milk, apple juice, and boneless chicken breasts were bought from local retailers while the water utilized was sterile municipal faucet water. Briefly, 8 log_10_ PFU/mL of the isolated pure phage was mixed with sterile milk, water, and apple juice, the same titer was spotted on the surface of sterile chicken breast pieces (1 cm^2^). The phage-treated food samples were then incubated for 48 h at either 4°C or 25°C, and aliquots were taken at 0, 2, 6, 12, 16, 24, 36, and 48 h, and phage titers were enumerated using DLA method.

#### Biological control of *Salmonella* in foods using phage LPSent1

The efficacy of applying phage LPSent1 as a biocontrol against *S.* Enteriditis EG.SmE1 in the above-mentioned food matrices was performed by measuring the recovered *Salmonella* load post-phage treatment as described previously (Huang et al., 2018b; Islam et al., 2019; Esmael et al., 2021b). To assay on milk, water, and apple juice, 10 μL of fresh *S.* Enteriditis EG.SmE1 (final count of 5 log_10_ CFU/mL) was added individually to each food article, then the phage was added at a MOIs of 100 or 1000. Chicken breast slices (1 cm^2^ each) were infected with *S.* Enteriditis EG.SmE1 (5 log_10_ CFU/mL), slices were air-dried for 30 min., and subsequently, were dipped in the phage solutions at MOIs of 100 or 1000. Equivalent volumes of SM buffer were added instead of the phage for the negative control treatments. for 48 h at either 4°C or 25°C, aliquots were taken at 0, 2 h, 6 h, 12 h, 16 h, 24 h, 36 h, and 48 h to count the recovered bacteria as explained previously (Islam et al., 2019).

### Statistical analysis

Student’s t-test was used to determine the significance of differences; P-values of ≤ 0.05 were considered significant.

## Results and discussion

### Antibiotic sensitivity testing and biofilm formation

The universal infection problem caused by *Salmonella* spp. denotes a considerable fraction of bacterial infections developed by the consumption of polluted food and water. The World Health Organization declared *Salmonella* is 1 of 4 key global reasons of diarrheal illnesses, and almost 10^6^ human cases caused by *Salmonella* spp. in the U.S. are foodborne (Scallan et al., 2011). The antibiotic sensitivity examination of the five isolates of *Salmonella* enterica serovars, previously isolated from a poultry farm in Egypt, was estimated against a range of thirteen antibiotics belonging to 10 different classes (Table 1).

**TABLE 1:** Antibiotic susceptibility pattern of five *Salmonella enterica* serovars against a selection of thirteen antibiotics.

Antibiotic category	Antibiotic tested	*S*. Enteritidis		*S*. Typhimurium		
		**EG.SmE1**	**EG.SmE2**	**EG.SmT1**	**EG.SmT2**	**EG.SmT3**
Penicillins	Ampicillin (10 μg)	R	S	S	S	R
	Amoxicillin (25 μg)	R	S	R	I	I
Fluoroquinolones	Ciprofloxacin (5 μg)	R	S	S	S	R
Aminoglycosides	Amikacin (30 μg)	I	S	S	R	S
	Gentamycin (10 μg)	I	R	I	S	R
	Streptomycin (10 μg)	R	S	R	I	S
Tetracyclines	Tetracycline (30 μg)	R	S	S	S	R
Phenolics	Chloramphenicol (30 μg)	R	S	R	R	S
Monobactams	Aztreonam (30 μg)	S	S	S	S	S
Sulfonamides	Trimethoprim/sulfamethoxazole (25 μg)	R	R	S	R	R
1st generation cephalosporins	Cephalexin (30 μg)	R	R	R	R	R
2nd generation cephalosporins	Cefoxitin (30 μg)	I	S	S	R	S
3rd generation cephalosporins	Ceftriaxone (30 μg)	S	I	I	S	I
Percentage of resistance (%)		**61%**	**23%**	**30%**	**38%**	**53%**

Bacterial isolates were susceptible (S; green), intermediate (I; yellow), or resistant (R; red) to the tested antibiotics.

The antibiogram data identified that the five *Salmonella* isolates resisted at least 23% of the examined antibiotics, however, a high resistance percentage of 61.5% and 53% for *S.* Enteriditis EG.SmE1 and *S*. Typhimurium EG.SmT3 respectively. Remarkably, all the tested bacteria were identified as MDR because they resisted at least one antibiotic within more than three different classes. MDR *Salmonella* have been isolated previously from Egypt (Abdelhakim et al., 2011; Abdel-Maksoud et al., 2015; Mahmoud et al., 2018; Merwad and Abdel-Haliem, 2018; Esmael et al., 2021b; Abd-Elghany et al., 2022). In Egypt there are no boundaries on antibiotic remedies and antibiotics can be acquired easily without any medical prescription from drugstores and pharmacies (Sobhy et al., 2012; Sabry et al., 2014; Awad et al., 2016; Esmael et al., 2020). Moreover, the poultry industry is one of the main foundations of contamination with veterinary antibiotics into the surrounding environment (Dahshan et al., 2015), as a result, the emergence of MDR bacteria is unceasing very fast.

**FIGURE 1:**
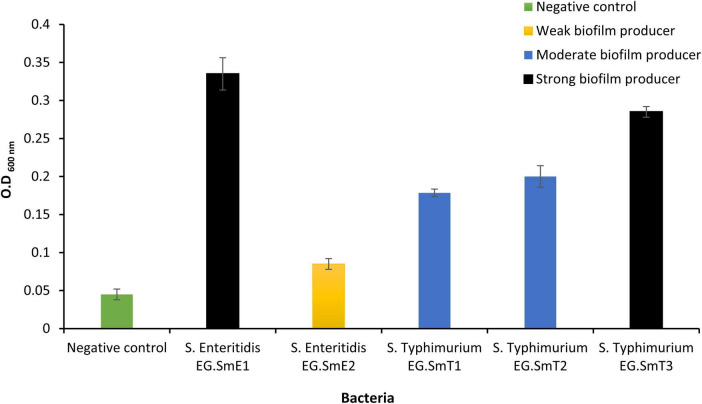
Quantitative evaluation of biofilm formation by five *Salmonella* serovars. Bacteria in the current study were classified into three categories; weak biofilm producers (*S*. Enteritidis EG.SmE2), moderate biofilm producers (*S*. Typhimurium EG.SmT1 and *S*. Typhimurium EG.SmT2), strong biofilm producer (*S*. Enteritidis EG.SmE1 and *S*. Typhimurium EG.SmT3). The negative control represents the optical density cutoff value. Data shown represent the average of three reads ± standard deviations.

Biofilms occur as cumulative bunches of densely populated bacteria that could be from single or multiple species. Biofilms of *Salmonella* help for the spread and persistence since they resist antibiotics, disinfectants, physical and chemical stresses (Humphrey, 2004; Spector and Kenyon, 2012) and lead to numerous foodborne infection outbreaks (Corcoran et al., 2014). The capability of the five *Salmonella* enterica serovars to form multicellular communities or biofilms was evaluated (Figure 1). The optical density of an uninoculated tryptone soy broth (negative control; bacteria-free medium) was used as a cutoff value (ODc) and was measured to be 0.06. Quantitative biofilm data generated by the five *Salmonella* serovars identified three categories based on their biofilm potency. One isolate, *S*. Enteritidis EG.SmE2, was identified as a weak biofilm producer (0.12 ≥ OD > 0.06). Two isolates, *S*. Typhimurium EG.SmT1 and *S*. Typhimurium EG.SmT2, were found to be moderate biofilm producers (0.24 ≥ OD > 0.12). Two isolates, *S*. Enteritidis EG.SmE1 and *S*. Typhimurium EG.SmT3, were identified as strong biofilm producers (OD > 0.24). Bacterial biofilms can be generated on food bodies, contaminated food utensils, and water. It is worth noting that the generation of bacterial biofilms, as a bacterial virulence factor, is of great concern as it has been linked to the increasing rates of antimicrobial resistance (Corcoran et al., 2014) which make the traditional treatment strategies not effective anymore.

### Isolation, purification, and screening of the candidate bacteriophages

The foregoing experiments revealed that *S*. Enteritidis EG.SmE1 and *S*. Typhimurium EG.SmT3 are extreme MDRs and strong biofilm producers. Traditional interference approaches for opposing MDR foodborne *Salmonella* are not effective enough to resolve the impasse of food quality and food safety. Hence, non-antibiotic biocontrol alternative therapies are of great interest to eradicate pathogenic bacteria from raw and retail food products. Bacteriophages are diverse and ubiquitous in every ecosystem with nearly 10^31^ virions on the earth (Rohwer and Segall, 2015; Othman et al., 2021; Abo-ELmaaty et al., 2022). Bacteriophages display a robust lytic efficacy against their bacterial host regardless of their antibiotic resistance pattern and can be a promising potential candidate to solve the dilemma (Esmael et al., 2021a,b; Teklemariam et al., 2023).

Previously we isolated three lytic bacteriophages targeting *S*. Typhimurium EG.SmT3 and the efficacy of those phages to combat *Salmonella* in food were established (Esmael et al., 2021b), therefore, *S*. Enteritidis EG.SmE1 was targeted in the current study as a reference host for the isolation of lytic bacteriophages. *S*. Enteritidis EG.SmE1 was prophage-free as validated using a mitomycin-C induction experiment, which advocated that it is an appropriate isolation host for lytic phages.

**FIGURE 2:**
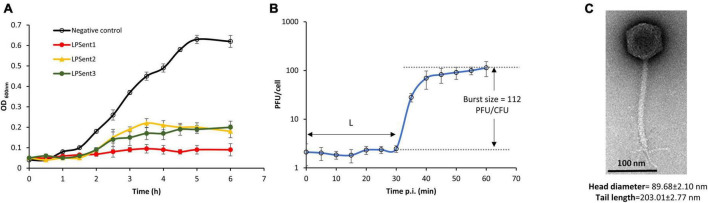
**(A)** Lysis ability comparison of the three isolated phages on *S*. Enteritidis EG.SmE1 as a host at an MOI of 1 in TSB. **(B)** Growth-kinetics of phage LPSent1 on *S*. Enteritidis EG.SmE1, phage burst size was calculated as 112 PFU/cell, L refers to the latent period. **(C)** Transmission electron micrograph of phage LPSent10, scale bar = 100 nm. Data shown in **(A,B)** are the mean of three replicates and error bars show the standard deviation in the values.

A total of 3 lytic bacteriophages were effectively isolated from three different sites; phages LPSent1 and LPSent2 were isolated from raw sewage water, while phage LPSent3 was isolated from a wastewater treatment plant. The three isolated phages showed remarkable discrepancies in plaque sizes and morphology. Phage LPSent1 formed a larger and clear plaque with distinct edges with a diameter of 1 mm. Likewise, phages LPSent2 and LPSent3 formed clear plaques of a diameter of 0.6 and 0.5 mm, respectively. To pick out the highly efficient phage, lytic activity was evaluated against *S*. Enteritidis EG.SmE1, as shown in Figure 2A. The data indicated that the three phages had inhibited *S*. Enteritidis EG.SmE1 growth 2 h p.i.; but, phages LPSent2 and LPSent3 lost their activity by 2.5 h p.i. Phage LPSent1 was robust and maintained a stable lytic activity, therefore, it was selected for further analysis.

### Characterization of phage LPSent1

In order to make the most of the benefits of bacteriophage-based applications, the cautious choice of the applicant phages is the primary point in the course and is vital for their effective application to improve food protection. We characterized phage LPSent1 to tailor the treatment or the application protocol. In addition, it emphasizes that phage LPSent1 does not have any resistance or pathogenic traits to the inhabitant human microbiota.

### Growth curve and TEM morphology

To examine phage LPSent1 infection cycle, a one-step growth curve was performed as shown in Figure 2B. Growth kinetics at MOI of 1 indicated a short latent period of 30 min and the phage required about 60 min to complete its infection cycle with an average burst size of 112 phages per single infected bacterium. Parameters like latency period and burst size have been formerly defined as appropriate to depict the lytic capacity of a phage (Rivera et al., 2022).

TEM examination showed that phage LPSent1 had an isometric capsid along with a contractile tail, with fixation structures (Figure 2C). Phage LPSent1 had an icosahedral head with a diameter of 89 ± 2.10 nm, the tail length was around 203.01 ± 2.77 nm. These results proposed that it has a siphovirus-like morphotype with a characteristic icosahedral head, collar, long non-contractile tail, tail fibers, base plate, and a spike.

### Thermal and pH tolerance

**FIGURE 3:**
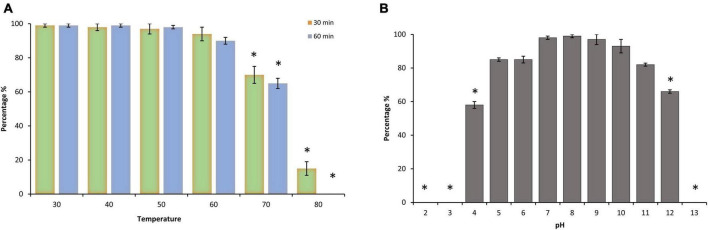
Thermal and pH stability test of phage LPSent1. **(A)** Thermal stability of LPSent1. **(B)** pH stability of LPSent1. Temperature stability tests were performed at 40, 50, 60, 70, and 80°C for 30 min or 60 min at pH 7, the control temperature was set at 30°C for 30 min or 60 min. pH experiments were performed at pH range 2-13 for 24 h at 37°C, pH 7 represented the control value around which the deviations were measured. The data (*n* = 3) displayed the percentages of the residual phages after each treatment, as normalized from the control ± standard error and were analyzed by Student’s *t*-test; *indicates significantly different from the controls, *p* ≤ 0.05.

Environmental stability is a limiting factor that confers an advantageous criterion of a phage and defines the aptitude to endure efficacy if extra heat or pH is to be added at any step during the decontamination process. Thermal and pH tolerance of phage LPSent1 was calculated by measuring the residual phage titer after incubation under a range of temperature and pH values (Figure 3). Phage LPSent1 is a thermostable bacteriophage with a tolerance range from 30°C to 70°C (Figure 3A). Phage LPSent1 significantly lost about 85% of its initial titer when heated at 80°C for 30 min. No viable LPSent1 plaques were recovered upon heating at 80°C for 60 min.

Phage LPSent1 also exhibited a high degree of pH stability throughout a pH range of 4-12 for 24 h (Figure 3B). LPSent1 titers were significantly dropped totally at extremely acidic or alkaline pH values, as no visible plaques were recovered at pH < 4 or > 12. Earlier studies displayed that most lytic bacteriophages are highly stable at pH < 4 or > 9 (Chow and Rouf, 1983; Woo and Ahn, 2014; Jung et al., 2017). As a result, phage LPSent1 can be applied in a wide range of foods due to its broad thermal stability and pH stability.

### Host range

The host range of phage LPSent1 was evaluated using undiluted and diluted phage stocks against a group of 18 *Salmonella* strains and 6 non-*Salmonella* strains as shown in Table 2. Spotting undiluted phage on the tested bacteria resulted in clear or turbid zones in 13 strains of the tested *Salmonella* strains (*n* = 18). Out of those 13, only 9 strains showed remarkable plaques when challenged with the diluted phage stock. The formation of individual plaques, using diluted phage stock, confirmed the susceptibility of 9 strains to phage LPSent1. However, the strains that initially showed turbidity or clear zones, when challenged with the undiluted phage stock, but could not form individual plaques, with diluted phage stock, were likely due to toxic bacteriocins produced during phage preparation or because of the disruption of bacterial cell membranes (Rendueles et al., 2022). Neither the undiluted nor the diluted phage stocks preparations broke the boundary of the genus as they could not lyse any of the 6 non-*Salmonella* strains tested.

**TABLE 2:** The host range of phage LPSent1 against different bacteria.

Species	Isolate ID number^1^	Undiluted phage^2^	Diluted phage^3^	EOP^4^
*S.* Enteritidis	EG.SmE1 (Enrichment host)	Clear	Plaques	Host
	EG.SmE2	Clear	Plaques	1
	EG.SE1	Clear	Plaques	0.95
	331SM	Clear	Plaques	0.77
	ShmE1	Clear	Plaques	0.67
*S.* Typhimurium	EG.SmT1	Turbid	–	NA
	EG.SmT2	Clear	plaques	0.05
	EG.SmT3	Clear	plaques	0.27
	101SM	Clear	plaques	0.05
	Shm1	Turbid	–	NA
	Shm2	Not clear	NA	NA
*S.* Kentucky	AE7	Turbid	–	NA
	AE12	Turbid	plaques	0.1
	AE51	Turbid	–	NA
S. Typhi	SamTph1	Not clear	NA	NA
	SamTph2	Not clear	NA	NA
	SamTph5	Not clear	NA	NA
*S. para* Typhi	102	Not clear	NA	NA
*E. coli*	BE1	Not clear	NA	NA
	BE2	Not clear	NA	NA
	BE3	Not clear	NA	NA
*S. aureus*	SA101	Not clear	NA	NA
	SA1E	Not clear	NA	NA
	EG-AE1	Not clear	NA	NA

^1^Supplementary file: Supplementary Table 1 displays the source of the bacterial isolates.

^2^Spot test made with undiluted phage stock (about 1 × 10^7^ PFU). Results were estimated as “Not clear” as no inhibition zone was detected; “Clear” as complete lysis was detected; “Turbid” as the inhibition zone was particularly turbid.

^3^Spot tests were performed with diluted phage stock (up to 10^7^ dilutions) for isolates that showed either turbid or clear lysis with the undiluted phage stock. Results were estimated as “plaques” as the development of plaques is indicated; “-” means no plaques were formed; “NA” as isolates that were not tested.

^4^Efficiency of plating (EOP) of phage LPSent1 on isolates that showed plaques formation. EOP 0.5-1.0, high efficiency; EOP 0.2 to < 0.5, moderate efficiency; 0.001 to < 0.2, low efficiency; and < 0.001, inefficient.

The efficiency of plating (EOP) of phage LPSent1 was calculated for the 9 susceptible strains that formed visible plaques as shown in Table 2. Other than the reference host, phage LPSent1 showed a high efficiency (0.5-1) against all the tested *S*. Enteritidis strains but moderate to low EOP was detected against *S*. Typhimurium or *S*. Kentucky strains. The results showed that phage LPSent1 had a robust lytic activity with a wide host range against several strains of *S*. Enteritidis, *S*. Typhimurium and *S*. Kentucky. Bacteriophage Felix 01 is a good example of a wide host range; it lyses nearly 98.5% of the tested *Salmonella* (Welkos et al., 1974). However, using a phage cocktail might solve the limitation of a narrow host range bacteriophages.

### Phage genomic characterization

**FIGURE 4:**
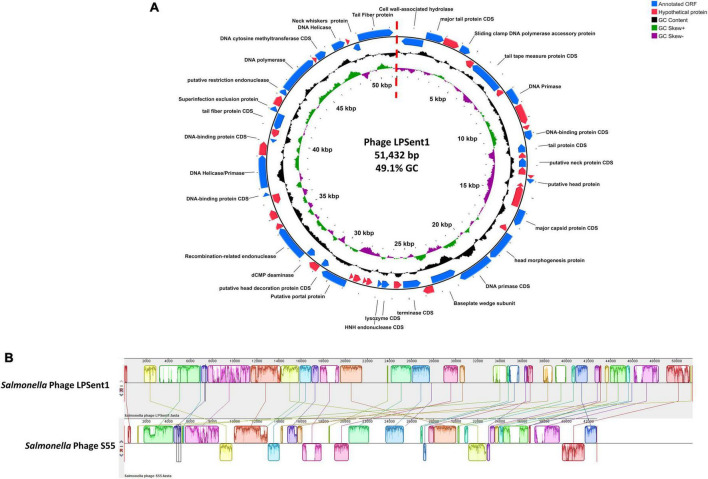
Phage LPSent1 whole genome analysis. **(A)** The LPSent1 genome organization represented as a circle. The 59 CDSs are represented as blue or red arrows showing the predicted genes transcribed clockwise (outer side) and counterclockwise (inner side) along the genome. The genome map is a circle, but there is a break at the 12 o’clock position (at the dotted straight red line) because the phage genome is a linear molecule. CDSs with known functions (blue arrows) are labeled with their putative functions along with their position; however, other non-labeled CDSs represent hypothetical proteins (red arrows). **(B)** Multiple alignment of phage LPSent1 with the most similar phage (phage S55) using progressive mauve alignment on default settings for *Salmonella* phage LPSent1 (top) and *Salmonella* phage S55 (bottom). The degree of DNA sequence similarity is indicated by the height of each colored block. Homologous regions are connected by lines between genomes, and blocks below the center line in *Salmonella* phage S55 indicate sequences with inverse orientation in comparison to phage LPSent1 arrangement. White spaces within blocks represent small-localized areas of the genome sequences that were not aligned.

The Illumina NextSeq 2000 was used to sequence a dsDNA genome of 51,432 bp for phage LPSent1 and it is displayed in a linear topology in Figure 4A. Most of the sequenced phages in the genebank databases have linear dsDNA genomes and are members of the order *Caudovirales* (Hatfull and Hendrix, 2011).

Analysis of the nucleotide composition of phage LPSent1 indicated a G + C content of 49.1% and a total of 57 putative open reading frames (ORFs) were identified (using the standard genetic code and ATG as initiation codon). Thirty ORFs are located on the leading strand and 27 ORFs are on the complementary strand, the minimum and maximum lengths of ORFs are 177 and 2,463 bp, respectively. BLASTp search annotated 26 ORFs encoded putative hypothetical proteins with unknown functions, while 31 ORFs wew homologus to functional proteins in the GenBank database in which have a high identity to annotated proteins of phages in *Jerseylikevirus* the genus.

The LPSent1 genome functionally encodes genes that include DNA replication/regulation, packaging, host cell lysis, and structural proteins (Supplementary Table 3 and Figure 4A). Phage LPSent1 did not encode any virulence genes, integrases, antimicrobial-resistant genes, or lysogenic genes which is a chief principle for phages proposed to be used in biocontrol applications (Omwandho and Kubota, 2010; Mahony et al., 2011; Esmael et al., 2021b). Previous studies reported the efficacy of lytic phages in the *Siphoviridae* family to combat different *Salmonella* serovars (Huang et al., 2018b). These indications specify that phage LPSent1 is considered a potentially promising candidate to biocontrol the MDR *S*. Enteritidis EG.SmE1 in different food matrices.

### Phage phylogenetic analysis

To identify the relationship between phage LPSent1 with other phages, BlastN comparison search of the non-redundant database at the NCBI was accomplished. LPSent1 genome sequence identified a 73% nucleotide homology (Figure 4B and Supplementary Table 2) with the previously sequenced *Salmonella* phage S55 belonging to the genus *Jerseyvirus* within the *Jerseyvirinae* subfamily.

**FIGURE 5:**
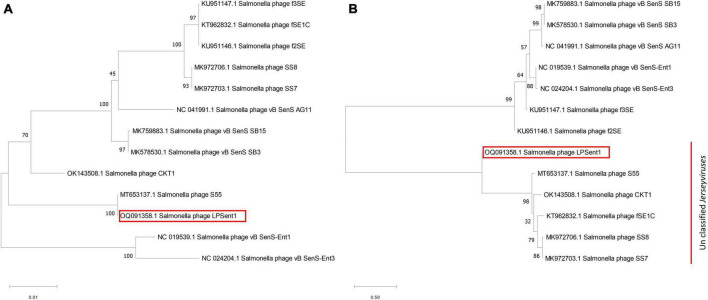
Neighbor-joining phylogenetic tree based on **(A)** terminase large subunit gene, **(B)** complete genome sequences of phage LPSent1 and related phages in *Jerseyviruses.* Numbers are shown next to the branches indicated by the percentage of replicate trees of the bootstrap test (1000 replicates).

Neighbor-joining (NJ) phylogenetic trees between phage LPSent1 and 12 additional phages in the *Jerseyvirus* genus were constructed based on (a) terminase large subunit (b) whole genome sequence comparisons in order to further examine and confirm the taxonomic rank of phage LPSent1. The phylogenetic tree constructed using the terminase large subunit gene (Figure 5A) showed that phage LPSent1 was closely linked to that of *Salmonella* phage S55 (MT653137.1). However, the phylogenetic tree inferred relying on the whole genome sequences (Figure 5B) showed the formation of an isolated phylogenetic position of phage LPsent1 within the unclassified *Jerseyvirus* clade. The phylogenetic analyses supported that phage LPsent1 is a novel species within the *Jerseyvirus* genus.

### Efficacy of phage LPSent1 on the planktonic cells and biofilms of *S*. Enteriditis EG.SmE1

**FIGURE 6:**
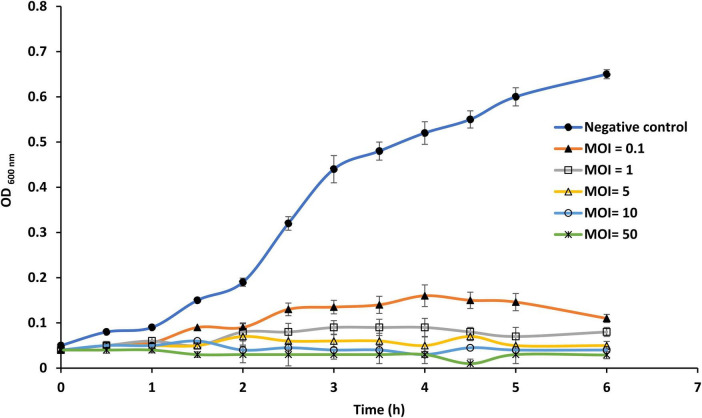
*In vitro* virulence of phage LPSent1 on free planktonic cells of *S*. Enteritidis EG.SmE1 at different multiplicity of infections (MOIs). The lysis activity of phage LPSent1 was evaluated at MOIs of 0.1, 1, 5, or 10. Bacterial cells were incubated for 24 h at 37°C, bacterial growth was monitored for 6 h by measuring optical density at 600 nm using a microplate reader (680 XR reader, Bio-Rad, Hercules, CA, USA). Data shown are the means of three replicates and error bars show the standard deviation in the values.

Susceptibility of planktonic cells of *S*. Enteritidis EG.SmE1 against phage LPSent1 was investigated at different MOIs for 6 h P.I. (Figure 6). The results showed that the efficacy of phage LPSent1 to inhibit the growth of planktonic cultures of *S*. Enteritidis EG.SmE1 is more robust in high MOIs as compared with the uninfected control. Additionally, the disruption lytic activity was retained for 6 h p.i. It is worth mentioning that the infection at all MOIs resulted in an extended lag phase up to 2 h post-infection, demonstrating that phage LPSent1 had an inhibition effect on *S*. Enteritidis EG.SmE1. Challenging with high phages MOIs increase the likelihood of phages to attach to the receptor sites on their hosts and accordingly enhance their lytic activity to eliminate large numbers of the host within a short time (Tanji et al., 2004; Bai et al., 2019; Esmael et al., 2021b).

The ability of *S*. Enteritidis EG.SmE1 to recover after initial infection at lower MOIs might be likely because of the emergence of bacteriophage-insensitive mutants (Guenther et al., 2012). One of the foremost worries of phage applications is the formation of Bacteriophage Insensitive (BIM) (Coffey et al., 2010). The frequency of *S*. Enteritidis EG.SmE1 to form BIM was assessed after incubation with phage LPSent1. The experiment showed that *S*. Enteritidis formed lower resistant mutants (1.34 × 10^–11^) against phage LPSent1, proposing that it might overcome the host-resistant mechanism (Labrie et al., 2010).

**FIGURE 7:**
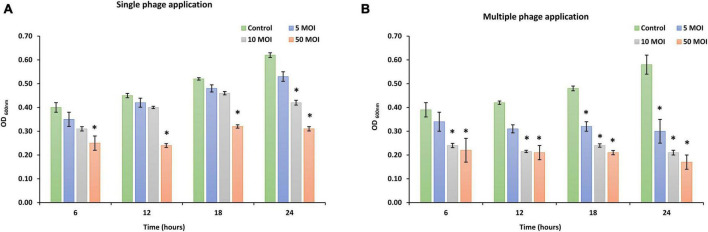
*In vitro* virulence of phage LPSent1 on biofilms of *S*. Enteritidis EG.SmE1 at different MOIs. **(A)** A single treatment with phage LPSent1. **(B)** Multiple treatments with phage LPSent1. Phage LPSent1 was applied at three different MOIs (5, 10, 50) while untreated cells were used as a negative control. Values represent the mean (*n* = 3) with a standard deviation of three determinations of each point and were analyzed by Student’s *t*-test; *indicates significantly different from the corresponding control, *p* ≤ 0.05.

The effectiveness of phage LPSent1 to disrupt surface-attached biofilm of *S*. Enteritidis EG.SmE1 was evaluated for 24 h (Figure 7) at different MOIs (5, 10, and 50). The quantity of viable *S*. Enteritidis EG.SmE1 cells (embedded in the biofilm-recovered solution) were reduced following either single or multiple phage applications. A single application of phage LPSent1 (Figure 7A) resulted in a significant inhibition in the established biofilm of *S*. Enteritidis EG.SmE1 following phage infection at 6 h. Interestingly, biofilm eradication rates were significantly higher when repeated applications of phage LPSent1 (Figure 7B) were used as compared to a single application. In addition, in either treatment, the higher the phage MOIs, the higher the biofilm eradication rate was detected, with an MOI of 50 displaying the highest reduction rates. Many previous studies advocate the efficiency of bacteriophages to eradicate *Salmonella* biofilms (Islam et al., 2019; Esmael et al., 2021b; Hosny et al., 2022; Mondal et al., 2022).

### Application of phage LPSent1 to control *S*. Enteritidis in contaminated food

**FIGURE 8:**
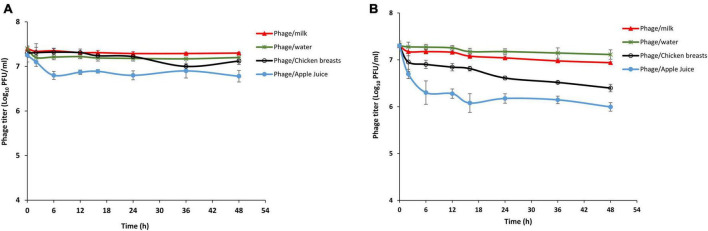
Analysis of LPSent1 stability in four food matrices (milk, water, apple juice and chicken breast). Phage-treated food samples were incubated for 48 h at either 4°C **(A)** or at 25°C **(B)**, and aliquots were taken at 0, 2, 6, 12, 16, 24, 36, and 48 h, and residual phage titers were measured as PFU/mL. Values represent the mean with a standard deviation of three determinations of each point.

Bacteriophages’ firmness in medium-lacking their susceptible hosts is a critical barrier in the fate of phage biocontrol applications. The stability of phage LPSent1 within food matrices including milk, potable water, chicken breasts, and apple juice was checked at both 4°C (the refrigerator storage temperature) and 25°C (room temperature at which the food is being administered or consumed) as shown in Figure 8. The data indicated that phage LPSent1 was retained in food with a small titer reduction within 48 h. However, phage LPSent1 titers dropped by roughly 0.9 and 1.5 log PFU/mL after 48 h incubation at 25°C on chicken breasts and in apple juice, respectively. We have previously shown the stability of a phage cocktail for 48 h at 4°C and 25°C in water, milk, and on chicken breast (Esmael et al., 2021b). Another study showed stability in phage titers for 24 h before it showed significant deterioration in cabbage and chicken breasts, however, better stability was shown in milk (Bao et al., 2015). The results indicated that phage LPSent1 was stable in the tested food samples and could be a potential promising candidate for controlling *Salmonella* in food.

**FIGURE 9:**
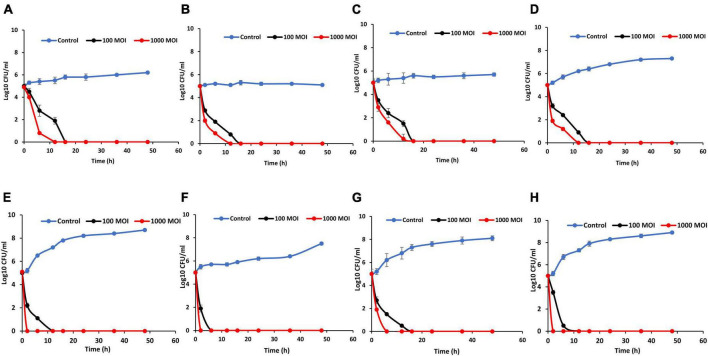
Effectiveness of phage LPSent1 in reducing the *S*. Enteritidis EG.SmE1 in milk, water, apple juice, and on chicken breasts at either 4°C or 25°C. **(A,E)** Application in milk at 4°C or 25°C respectively, **(B,F)** application in water at 4°C or 25°C respectively, **(C,G)** application in apple juice at 4°C or 25°C respectively, and **(D,H)** application on chicken breasts at 4°C or 25°C respectively. Phage LPSent1 was applied at two different MOIs (100 and 1,000) to each food article and incubated for 48 h at either 4°C or 25°C. Bacterial load was estimated at different time points by measuring the mean CFU/mL ± standard deviation of three replicates of each time point.

The influence of phage LPSent1 against the MDR *S*. Enteritidis EG.SmE1 contaminated food was assessed (Figure 9) at two different temperatures (4°C and 25°C). Food samples were contaminated with *S*. Enteritidis EG.SmE1 at a final count of 5 log_10_ CFU/mL and then challenged with the phage either at 100 or 1000 MOIs. In milk assay, the viable count of *S*. Enteritidis EG.SmE1 dropped below the detection threshold (< 1 CFU/100 μL) after 12 h and 16 h at 4°C challenging with phage LPSent1 at MOIs of 1000 and 100, respectively (Figure 9A). While at 25°C, no bacterial count was detected after 2h and 12 h at MOIs of 1000 and 100, respectively (Figure 9E). Previously, we showed the same inhibition values using a phage cocktail against the MDR S. Typhimurium EG.SmT3 (Esmael et al., 2021b).

Biocontrol in water was similarly investigated (Figures 9B, F). Relative to the non-treated controls *S*. Enteritidis EG.SmE1 counts declined below the detection limit and no viable counts were detected after 12 h and 16 h at 4°C post-phage infections using MOIs of 1000 and 100, respectively. *Salmonella* counts were dropped faster at 25°C, it only took 2 h and 6 h post-phage LPSent1 treatment at MOIs of 1000 and 100, respectively for the complete elimination of *Salmonella* in water. In the apple juice assay (Figure 9C), there were almost no viable counts of *S*. Enteritidis EG.SmE1 after 16 h at 4°C following treatment with LPSent1 at MOIs of 1000 and 100. While at 25°C, no viable bacterial count was detected after 6h and 16 h at MOIs of 1000 and 100, respectively (Figure 9G). Phage LPSent1 bestowed a noticeable reduction of viable *S*. Enteritidis EG.SmE1 counts on chicken breast slices at either 4°C or 25°C. When applied at 4°C using MOIs of 1000 and 100, phage LPSent1 reduced the viable bacterial counts below the detection limit after 12 h and 16 h, respectively (Figure 9D). In the case of application at 25°C (Figure 9H), no viable bacterial counts were detected after 2 h and 16 h after adding LPSent1 at MOIs of 1000 and 100, respectively.

Interestingly our results showed a promising reduction rate in the growth of the MDR *S*. Enteritidis EG.SmE1 in the tested food articles using phage LPSent1 for 2 days, especially when applied at 25°C, as compared with the phage-free controls. It is worth mentioning that, phages’ virulence is decreased at low temperatures as at low temperatures the bacterial host growth is hindered and hence the phage virulence is decreased (Bigwood et al., 2008; Guenther and Loessner, 2011; Huang et al., 2018b). Earlier studies reported a significant reduction rate of nearly 3 Log_10_ units in the recovered *Salmonella* Enteritidis using phage cocktails at MOIs of 1000, and 10,000 in different food matrices (Goodridge and Bisha, 2011; Kang et al., 2013; Spricigo et al., 2013; Bao et al., 2015; Grant et al., 2016; Osvaldo et al., 2016; Sharma et al., 2017; Islam et al., 2019; Esmael et al., 2021b).

## Conclusion

The current study explains the environmental isolation of a robust and lytic bacteriophage against an antibiotic-resistant pathogenic *S*. Enteritidis EG.SmE1. Phage LPSent1 is a lytic bacteriophage with robust pH and thermal stability. Phage LPSent1 efficiently reduced free planktonic cells and biofilms of the MDR *S*. Enteritidis EG.SmE1. The *in vitro* application of phage LPSent1 on artificially contaminated foods was assessed on milk, water, apple juice, and on chicken breast and showed a significant reduction of the recovered bacteria as compared with the untreated controls. Additionally, the genome of phage LPSent1 was sequenced and analyzed in the current study. Subsequently, the current work identifies phage LPSent1 as a promising potential bio-control agent in the food industry.

## Data availability statement

The datasets presented in this study can be found in online repositories. The names of the repository/repositories and accession number(s) can be found in the article/Supplementary material.

## Author contributions

RA-H contributed to the conception, design, project implementation, and manuscript revision. MA, IA, SA, and KA contributed to the conception and manuscript preparation and revision. AE contributed to the conception, design, project implementation, run the experiments, data interpretation, and wrote and revised the manuscript. All authors contributed to the article and approved the submitted version.
