# Supplementary material for: Application of a novel lytic Jerseyvirus phage LPSent1 for the biological control of the multidrug-resistant Salmonella Enteritidis in foods
Source: Front Microbiol. 2023 Apr 5;14:1135806. doi: 10.3389/fmicb.2023.1135806 (PMC10113451; doi:10.3389/fmicb.2023.1135806)
Supplement: Supplementary file 1 [file Table_1.DOCX]

Supplementary Material

**Application of a novel lytic *Jerseyvirus* phage LPSent1 for the biological control of the multidrug-resistant *Salmonella* Enteritidis in foods**

*Rashad R. Al-Hindi^1,*^, Mona G. Alharbi^1^, Ibrahim Alotibi^2^, Sheren A. Azhari^1^, , Khloud M. Algothmi^1^ and Ahmed Esmael^3,4,*^*

^1^ Department of Biological Sciences, Faculty of Science, King Abdulaziz University, Jeddah 21589, Saudi Arabia, ^2^ Health Information Technology Department, Applied College, King Abdulaziz University, Jeddah 21589, Saudi Arabia, ^3^ Botany and Microbiology Department, Faculty of Science, Benha University, Benha 13518, Egypt, ^4^ Nebraska Center for Virology, University of Nebraska Lincoln, Lincoln, Nebraska 68583, USA.

^*^ **Correspondence**: Rashad R. Al-Hindi ([rhindi@kau.edu.sa](mailto:rhindi@kau.edu.sa)) and Ahmed Esmael ([ahmed.esmael@fsc.bu.edu.eg](mailto:ahmed.esmael@fsc.bu.edu.eg), [a7medesmael@gmail.com](mailto:a7medesmael@gmail.com))

**Table S1.** Bacterial strains used in the current study.

| **Species** | **Isolate ID number^1^** | **Source** |
| --- | --- | --- |
| ***S.* Enteritidis** | EG.SmE1 (Enrichment host) | AE^1^ |
|  | EG.SmE2 | AE |
|  | EG.SE1 | FSCBU^2^ |
|  | 331SM | FSCBU |
|  | ShmE1 | FSCBU |
| ***S.* Typhimurium** | EG.SmT1 | AE |
|  | EG.SmT2 | AE |
|  | EG.SmT3 | AE |
|  | 101SM | FSCBU |
|  | Shm1 | FSCBU |
|  | Shm2 | FSCBU |
| ***S.* Kentucky** | AE7 | FSCBU |
|  | AE12 | FSCBU |
|  | AE51 | FSCBU |
| **S.** **Typhi** | SamTph1 | FSCBU |
|  | SamTph2 | FSCBU |
|  | SamTph5 | FSCBU |
| ***S. para* Typhi** | 102 | FSCBU |
| ***E. coli*** | BE1 | FSCBU |
|  | BE2 | FSCBU |
|  | BE3 | FSCBU |
| ***S. aureus*** | SA101 | FSCBU |
|  | SA1E | FSCBU |
|  | EG-AE1 | FSCBU |

^1^ AE (From a poultry farm with a history of diarrhea).

^2^ FSCBU (Microbiology lab, Botany and Microbiology Dept., Faculty of Science, Benha Univ., Egypt).

**Table S2.** BLASTn alignment of homologs phage sequences (at the GenBank database) with *Salmonella* phage LPSent1

| **Phage** | **Isolated place** | **Accession#** | **Length (bp)** | **E value** | **Query**  **coverage** |
| --- | --- | --- | --- | --- | --- |
| ***Salmonella* phage S55** | China | [MT653137.1](https://www.ncbi.nlm.nih.gov/nucleotide/MT653137.1?report=genbank&log$=nucltop&blast_rank=1&RID=GTJY3HR7016) | 42781 | 0.0 | 73% |
| ***Salmonella* phage Shelanagig** | USA | [MK931446.1](https://www.ncbi.nlm.nih.gov/nucleotide/MK931446.1?report=genbank&log$=nucltop&blast_rank=2&RID=GTJY3HR7016) | 42541 | 0.0 | 63% |
| ***Salmonella* phage SHWT1** | China | [MT740291.1](https://www.ncbi.nlm.nih.gov/nucleotide/MT740291.1?report=genbank&log$=nucltop&blast_rank=3&RID=GTJY3HR7016) | 41850 | 0.0 | 69% |
| ***Salmonella* phage NBSal006** | Peru | [MT677933.1](https://www.ncbi.nlm.nih.gov/nucleotide/MT677933.1?report=genbank&log$=nucltop&blast_rank=4&RID=GTJY3HR7016) | 41850 | 0.0 | 64% |
| ***Salmonella* phage CKT1** | China | [OK143508.1](https://www.ncbi.nlm.nih.gov/nucleotide/OK143508.1?report=genbank&log$=nucltop&blast_rank=5&RID=GTJY3HR7016) | 40923 | 0.0 | 69% |

**Table S3.** Genome annotation of the phage LPSent1

| **ORFs** | **Start** | **Stop** | **Length** | **Direction** | **Putative function** |
| --- | --- | --- | --- | --- | --- |
| ORF1 | 296 | 1711 | 1416 | Reverse | Cell wall-associated hydrolase |
| ORF2 | 1798 | 2919 | 1122 | Forward | Major tail protein |
| ORF3 | 2926 | 3909 | 984 | Forward | Hypothetical protein |
| ORF4 | 4044 | 4685 | 642 | Forward | Sliding clamp DNA polymerase accessory protein |
| ORF5 | 4750 | 5268 | 473 | Reverse | Hypothetical protein |
| ORF6 | 5270 | 7552 | 2283 | Reverse | Tail tape measure protein |
| ORF7 | 7578 | 7994 | 417 | Reverse | Hypothetical protein |
| ORF8 | 8014 | 9066 | 1053 | Forward | DNA primase |
| ORF9 | 9115 | 10374 | 1260 | Forward | Hypothetical protein |
| ORF10 | 10487 | 10759 | 273 | Forward | Hypothetical protein |
| ORF11 | 10830 | 11414 | 585 | Forward | DNA-binding protein |
| ORF12 | 11578 | 12138 | 561 | Reverse | Tail protein |
| ORF13 | 12154 | 12513 | 360 | Reverse | Hypothetical protein |
| ORF14 | 12513 | 13118 | 606 | Reverse | Putative neck protein |
| ORF15 | 13121 | 13630 | 510 | Reverse | Hypothetical protein |
| ORF16 | 13634 | 13822 | 189 | Reverse | Hypothetical protein |
| ORF17 | 13870 | 14157 | 288 | Forward | Putative head protein |
| ORF18 | 14214 | 14390 | 177 | Reverse | Hypothetical protein |
| ORF19 | 14387 | 15814 | 1428 | Reverse | Hypothetical protein |
| ORF20 | 15884 | 16933 | 1050 | Forward | Major capsid protein |
| ORF21 | 17157 | 17546 | 390 | Reverse | Hypothetical protein |
| ORF22 | 17594 | 19453 | 1860 | Forward | Head morphogenesis protein |
| ORF23 | 19545 | 21731 | 2187 | Forward | DNA primase |
| ORF24 | 21774 | 23426 | 1653 | Reverse | Baseplate wedge subunit |
| ORF25 | 23463 | 24116 | 654 | Forward | Hypothetical protein |
| ORF26 | 24143 | 25309 | 1272 | Reverse | Terminase |
| ORF27 | 25404 | 25910 | 507 | Reverse | Hypothetical protein |
| ORF28 | 26208 | 26696 | 489 | Reverse | Lysozyme |
| ORF29 | 26674 | 26964 | 291 | Reverse | HNH endonuclease |
| ORF30 | 26966 | 27247 | 282 | Reverse | Hypothetical protein |
| ORF31 | 27326 | 27703 | 378 | Reverse | Hypothetical protein |
| ORF32 | 27700 | 27924 | 225 | Reverse | Hypothetical protein |
| ORF33 | 28091 | 28579 | 489 | Reverse | Hypothetical protein |
| ORF34 | 28620 | 28829 | 210 | Reverse | Hypothetical protein |
| ORF35 | 28916 | 30550 | 1472 | Forward | Putative portal protein |
| ORF36 | 30388 | 30846 | 459 | Reverse | Putative head decoration protein |
| ORF37 | 30850 | 31503 | 654 | Forward | Hypothetical protein |
| ORF38 | 31530 | 32042 | 513 | Reverse | dcmp deaminase |
| ORF39 | 32102 | 34291 | 2190 | Forward | Recombination-related endonuclease |
| ORF40 | 34295 | 34657 | 363 | Forward | Hypothetical protein |
| ORF41 | 34951 | 35556 | 606 | Forward | Hypothetical protein |
| ORF42 | 35800 | 36459 | 660 | Reverse | Hypothetical protein |
| ORF43 | 36481 | 36720 | 240 | Forward | DNA-binding protein |
| ORF44 | 36743 | 36976 | 234 | Forward | putative UvsX-like protein |
| ORF45 | 37012 | 39069 | 2058 | Forward | DNA helicase/primase |
| ORF46 | 39072 | 39923 | 852 | Forward | Hypothetical protein |
| ORF47 | 39936 | 40181 | 246 | Reverse | DNA-binding protein |
| ORF48 | 40315 | 40839 | 525 | Forward | Hypothetical protein |
| ORF49 | 40846 | 41928 | 1083 | Reverse | Tail fiber protein |
| ORF50 | 41941 | 42261 | 321 | Forward | Superinfection exclusion protein |
| ORF51 | 42331 | 42981 | 651 | Forward | Hypothetical protein |
| ORF52 | 43125 | 43490 | 366 | Forward | Putative restriction endonuclease |
| ORF53 | 43497 | 45959 | 2463 | Forward | DNA polymerase |
| ORF54 | 45985 | 46221 | 237 | Forward | Hypothetical protein |
| ORF55 | 46226 | 46888 | 663 | Forward | DNA cytosine methyltransferase |
| ORF56 | 47384 | 48193 | 1296 | Forward | DNA helicase |
| ORF57 | 48288 | 48512 | 226 | Forward | Hypothetical protein |
| ORF58 | 48533 | 48979 | 447 | Reverse | Neck whiskers protein |
| ORF59 | 49044 | 51209 | 2166 | Forward | Tail Fiber protein |
